# Multimodal Emotion Recognition Using Modality-Wise Knowledge Distillation

**DOI:** 10.3390/s25206341

**Published:** 2025-10-14

**Authors:** Seonggyu Lee, Youngdo Ahn, Jong Won Shin

**Affiliations:** School of Electrical Engineering and Computer Science, Gwangju Institute of Science and Technology, Buk-gu, Gwangju 61005, Republic of Korea; lsqjin2022@gm.gist.ac.kr (S.L.); ayoungdo@gm.gist.ac.kr (Y.A.)

**Keywords:** multimodal emotion recognition, knowledge distillation, optimization imbalance phenomenon

## Abstract

Multimodal emotion recognition (MER) aims to estimate emotional states utilizing multiple sensors simultaneously. Most previous MER models extract unimodal representation via modality-wise encoders and combine them into a multimodal representation to classify the emotion, and these models are trained with an objective for the final output of the MER. If an encoder for a specific modality is optimized better than others at some point of the training procedure, the parameters for the other encoders may not be sufficiently updated to provide optimal performance. In this paper, we propose a MER using modality-wise knowledge distillation, which adapts the unimodal encoders using pre-trained unimodal emotion recognition models. Experimental results on CREMA-D and IEMOCAP databases demonstrated that the proposed method outperformed previous approaches to overcome the optimization imbalance phenomenon and could also be combined with these approaches effectively.

## 1. Introduction

Emotion recognition refers to the techniques used to estimate emotional attributes or categorical emotions from various modalities such as speech, text, and video [1,2,3,4,5] captured by multiple sensors such as microphones and cameras, which can be applied to health care [6], automobility systems [7], and human–computer interactions [8]. Recently, multimodal emotion recognition (MER) has gained attention [9,10,11,12,13,14,15,16,17,18] as it mimics human perception of emotion utilizing multiple senses such as vision, hearing, and linguistic comprehension [19,20,21], and the performance of unimodal emotion recognition was not satisfactory. Although there are a few works on emotion recognition in conversation using contextual information [22,23,24,25], most of the research has focused on utterance-level emotion recognition, which estimates emotional state given an utterance [9,10,11,12,13,14,15,16,17,18]. The typical configuration of a MER model consists of unimodal encoders, which extract emotional representations from each modality; a fusion module, which combines them to produce multimodal representations; and a classifier to produce final probabilities for emotional classes. Usually, a single loss on the final output of the multimodal emotion classifier is used to train these models.

In [15], an analysis of the optimization imbalance phenomenon is provided, with an example for a simple fusion module and a linear classifier, in which the parameters of the unimodal encoders may not be sufficiently updated in training once an encoder for another modality produces more discriminative embeddings during the training procedure.

To facilitate the learning of less-optimized encoders, on-the-fly gradient modulation with generalization enhancement (OGM-GE) was proposed in [15], which penalizes the learning rate for the parameters of the more-optimized unimodal encoder. In the same context, a prototypical modal rebalance (PMR) method is proposed in [16], which introduces separate loss terms for individual modalities using the “prototype” features for each class and entropy regularization to slow the learning of the more-optimized encoder. The authors of [17] proposed a modality-wise cosine similarity (MMCosine) loss function, which normalizes the weighted embeddings from different modalities so that one modality cannot dominate the loss function.

These approaches provide several ways to mitigate the optimization imbalance among modalities, but the loss functions for training the MER models only apply to the final MER output. In [26], an ensemble of unimodal models and a multimodal model is presented, in which a portion of the parameters is shared across different unimodal models, but the multimodal model in this approach is also trained with the loss on the final MER output. In this paper, we propose a MER with a loss function on the MER output and unimodal representations for all modalities. To help with the optimization of each unimodal encoder, we employ a knowledge distillation (KD) technique [27,28] so that the pre-trained unimodal emotion classification models guide the training of the corresponding unimodal encoders within the MER model. Experimental results on the utterance-wise emotion classification using a microphone and camera sensors show that the proposed MKD approach outperformed the previously proposed methods for MER and can be combined with previous methods to further improve the results.

## 2. Method

### 2.1. Typical Multimodal Emotion Recognition Model and Optimization Imbalance Phenomenon

**Figure 1:**
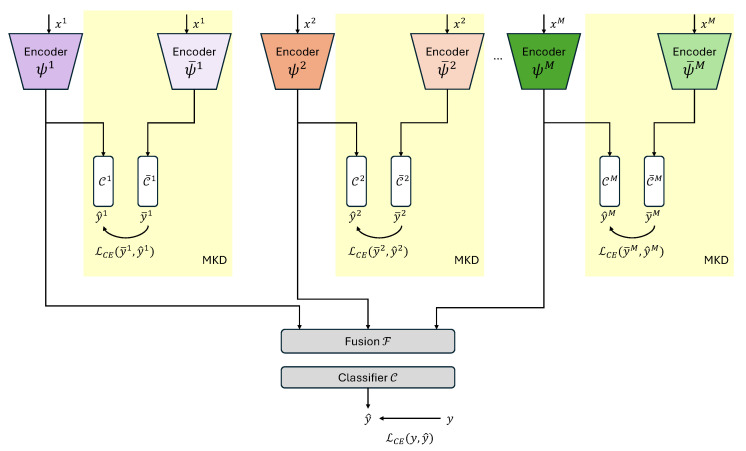
The overall block diagram for the proposed multimodal emotion recognition using modality-wise knowledge distillation (MKD). Yellow shaded blocks are used in the training phase only.

A MER model aims to classify emotion in multimodal input data $x={\left\{x^{m}\right\}}_{m=1}^{M}$, where $x^{m}$ represents the input feature for the *m*-th modality, and *M* is the number of modalities. Typically, a MER model *f* consists of unimodal encoders ${\left\{\psi^{m}\right\}}_{m=1}^{M}$, a fusion module F, and a classifier C, as shown in Figure 1.

Each encoder transforms the input features into unimodal representations, and the fusion module combines them into a multimodal representation. Then, the classifier maps the multimodal representation into a *C*-dimensional vector, where *C* is the number of emotion classes. Then, the output of the model can be described as

(1) $$f\left(x\right)=C\left(F\left(\psi^{1}\left(x^{1}\right),\psi^{2}\left(x^{2}\right),\ldots ,\psi^{M}\left(x^{M}\right)\right)\right).$$

The most common choice for the loss function is CE loss:

(2) $$L_{\mathrm{CE}}\left(y,\hat{y}\right)=-y\cdot \log\hat{y},$$

where *y* is a one-hot vector representing the emotion label of the input sample *x*, and $\hat{y}=\mathrm{softmax}\left(f\left(x\right)\right)$ is the logit from the MER model. In the simplest example in which F is a concatenation of $\psi^{m}\left(x^{m}\right)\begin{array}{c} \in R^{d_{m}} \end{array}$ and C is a linear classifier with weight matrices $W^{m}\in R^{C\times d_{m}},m=1,\cdots ,M$ and bias $b\begin{array}{c} \in R^{C} \end{array}$, (1) can be represented as

(3) $$f\left(x\right)=\sum_{m=1}^{M}\left(W^{m}\psi^{m}\left(x^{m}\right)+b/M\right)$$

where $W^{m}\psi^{m}\left(x^{m}\right)+b/M$ corresponds to the output of a classifier when only the *m*-th modality is present. It was reported in [15,17] that the elements of $W^{m}$ for a modality with a less-optimized encoder become small when one unimodal encoder is better optimized at some point during the training procedure if CE loss is applied to the MER output, disrupting the further optimization of the other encoders. There have been a few approaches to this optimization imbalance phenomenon, including the adjustment of the learning rate [15] and the use of modality-wise cosine similarity [17]. In this paper, we propose a different approach that can also be combined with the previously proposed methods, which is to adapt each unimodal encoder using the knowledge distillation from the pre-trained unimodal emotion recognition model.

### 2.2. Modality-Wise Knowledge Distillation

The overall block diagram of the proposed MER system employing modality-wise knowledge distillation to facilitate the optimization of all unimodal encoders is shown in Figure 1. We use a pre-trained unimodal emotion recognition model ${\bar{f}}^{m}$ to guide the unimodal encoder in the MER model $\psi^{m}$. Any unimodal emotion recognition model can be used as ${\bar{f}}^{m}$, but if it has the structure of unimodal encoder $\bar{\psi }$, followed by a linear classifier $\bar{C}$, it can be represented as

(4) $${\bar{f}}^{m}\left(x^{m}\right)={\bar{C}}^{m}\left({\bar{\psi }}^{m}\left(x^{m}\right)\right).$$

An additional classifier for each unimodal encoder in the MER model, $C^{m}$, is used for MKD to compare the emotion classification capability of the encoder with that of the pre-trained unimodal emotion recognition model ${\bar{f}}^{m}$. In the experiment, ${\bar{C}}^{m}$ and ${\bar{\psi }}^{m}$ are configured to have the same structure but different parameter values from $C^{m}$ and $\psi^{m}$, respectively. The target label vector ${\bar{y}}^{m}$ obtained from the unimodal pre-trained model is given by

(5) $${\bar{y}}^{m}=\mathrm{softmax}\left(\frac{{\bar{f}}^{m}\left(x^{m}\right)}{T_{m}}\right),$$

where $T_{m}$ is a hyperparameter called temperature. Then, the objective function for MKD is given by

(6) $$L_{\mathrm{MKD}}\left({\left\{{\bar{y}}^{m},{\hat{y}}^{m}\right\}}_{m=1}^{M}\right)=\sum_{m=1}^{M}\left[\lambda_{m}L_{\mathrm{CE}}\left({\bar{y}}^{m},{\hat{y}}^{m}\right)\right],$$

where ${\hat{y}}^{m}=\mathrm{softmax}\left(C^{m}\left(\psi^{m}\left(x^{m}\right)\right)\right)$ is the logit from classifier $C^{m}$ for the *m*-th unimodal representation, and *λ*_*m*_s are the hyperparameters for the weights. For each iteration in training, we first update $\psi^{m}$ and $C^{m}$ using the MKD loss in (6), and then we update the whole model’s parameters with the same minibatch using the MER loss, such as the CE loss in (2). In this way, each unimodal encoder has a chance to be updated according to the guidance of the corresponding pre-trained model and thus will not be less-optimized. In the inference phase, only MER model *f* is used, while $C^{m}$, ${\bar{C}}^{m}$, and ${\bar{\psi }}^{m}$ do not need to be kept.

### 2.3. Combination with Other Regularization Methods

We can combine MKD with previously proposed regularization methods [15,17]. In OGM-GE [15], which can be applied only for bimodal applications with M=2, the learning rates for two modality encoders are adjusted based on the discrepancy between the contributions of the modalities to the final output. colorredThe discrepancy $\rho_{t}^{m}$, where m∈{1,2} for the *t*-th minibatch $B_{t}$, is computed as

(7) $$\rho_{t}^{1}=\sum_{i\in B_{t}}\frac{s_{i}^{1}}{s_{i}^{2}},\;\;\rho_{t}^{2}=1/\rho_{t}^{1},$$

where $s_{i}^{m}$ for the *i*-th sample $x_{i}^{m}$ with one-hot label vector $y_{i}$ is

(8) $$s_{i}^{m}=y_{i}\cdot \mathrm{softmax}\left(W^{m}\psi^{m}\left(x_{i}^{m}\right)+b/2\right).$$

Then, a scaling factor $k_{t}^{m}$ is applied to the learning rate for the parameters regarding modality *m*:

(9) $$\begin{array}{c} k_{t}^{m}=\left\{\begin{array}{cc} 1-\tanh(\alpha \rho_{t}^{m}),\; & \mathrm{if}\;\rho_{t}^{m}>1,\\ 1,\; & \mathrm{otherwise}, \end{array}\right. \end{array}$$

where α>0 is a hyperparameter. For generalization enhancement, the parameter updates are perturbed by Gaussian noises with appropriate covariance matrices. In [17], MMCosine loss was adopted instead of CE loss so that one modality could not dominate the loss function. The MMCosine loss is given by

(10) $$L_{\mathrm{MMCosine}}\left(y,x\right)=-\log\frac{\exp\left(s\sum_{m=1}^{M}\cos\theta_{k}^{m}\right)}{\sum_{j=1}^{C}\exp\left(s\sum_{m=1}^{M}\cos\theta_{j}^{m}\right)},$$

where $\cos\theta_{j}^{m}$ $=\frac{W_{j}^{m}\psi^{m}\left(x^{m}\right)}{∥W_{j}^{m}∥∥\psi^{m}\left(x^{m}\right)∥}$ in which $W_{j}^{m}$ is the *j*-th row of $W^{m}$, s>0 is a hyperparameter, and *k* is the index of the emotion label for the current input. MMCosine loss can be used for an arbitrary number of modalities, unlike OGM-GE [15]. The training procedure combining MKD with other regularization methods is described in Algorithm 1.

**Algorithm 1:** MKD with optional imbalance mitigation methods (OGM-GE or MMCosine)

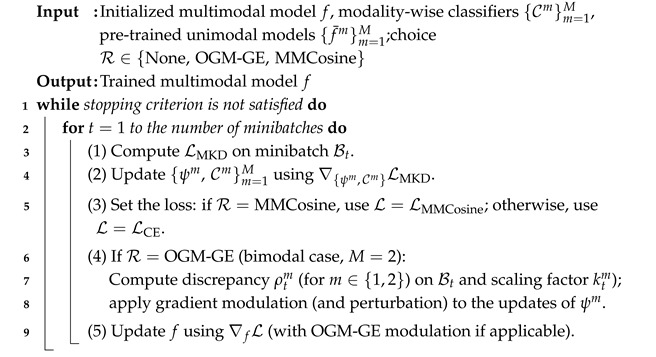



## 3. Experiments

### 3.1. Experimental Configurations

To demonstrate the performance of MKD in utterance-wise emotion recognition, two multimodal emotional databases were employed, which were the audio–visual dataset CREMA-D [29] and the audio–visual–text dataset IEMOCAP [30]. CREMA-D consists of 7422 video clips, which were recorded by 91 professional actors expressing pre-determined emotions for 12 pre-defined sentences. A total of 6698 clips were used as the training set, while 744 samples comprsied the test set. We considered six emotion classes for this dataset, which were angry, happy, sad, neutral, disgust, and fear. IEMOCAP is a dyadic conversation dataset performed by 5 male and 5 female actors including both scripted scenarios and improvisations. Three annotators assigned the emotion class labels to each speech utterance in IEMOCAP. We used 7 classes including angry, excited, happy, sad, frustrated, surprised, and neutral, and the number of utterances was 7487 [14]. We performed 5-fold cross-validation, dividing the data into a training set, validation set, and test set in an 8:0.5:1.5 ratio, as in [9,14,31]. We trained the model 10 times with different random initializations and report the performance averaged over 10 random seeds and 5-fold cross-validation for the IEMOCAP dataset, as in [9,14,31].

As for the features and model structures, we followed the configurations in [15] and [14] for CREMA-D and IEMOCAP, respectively. For CREMA-D, we used one frame sampled from a video clip as a visual feature and a spectrogram of 257×299 as an audio feature. For IEMOCAP, 300-dimensional GloVe embeddings of transcriptions were employed as text features. Also, 40-dimensional MFCC features with their first and second derivatives were used as the audio features, while 2048-dimensional Resnet-101 embeddings of a cropped face image at 3 Hz frame rates were utilized as the visual features. For the model structure, we used ResNet18 as the encoder for CREMA-D, as in [15], and the fusion was made by a simple concatenation, which outperformed other fusion methods such as sum, FiLM [32], and Gated [33]. For IEMOCAP, we utilized the tri-modal self-attention model in [14], in which the encoders contained convolution layers, bi-directional GRU, and multi-head attention layers. The outputs of three encoders were fused as in [14], where the encoder outputs for frames were temporally averaged first, and then the mean and the standard deviations of the temporal averages for three modalities were concatenated to form the fused vector.

The performance of the proposed MKD was compared with that of the original MER systems in [14,15], using previous approaches for mitigating the optimization imbalance phenomenon including OGM-GE [15], PMR [16], and MMCosine [17], the ensemble of unimodal emotion recognition models (denoted Uni-sum), and the ensemble of unimodal and multimodal emotion recognition models (denoted All-sum) [26]. To verify the performance improvement was from the modality-wise KD rather than KD itself, we implemented Self-KD [34] in which the softmax output of a pre-trained MER model with the same structure provided the target for the MER output. We also tested the use of modality-wise CE loss (MWCE) instead of MKD loss using the ground-truth one-hot label vector to show that MKD was indeed effective compared with guiding the unimodal encoders with additional losses. Additionally, we applied MKD along with OGM-GE [15], PMR [16], and MMCosine [17], and we tested the ensemble of MKD and unimodal models (denoted All-sum (MKD)). It is noted that OGM-GE and PMR are essentially bimodal methods and thus were not tested for IEMOCAP in our experiment.

For the models applied to CREMA-D, the learning rate schedule and the stopping criterion followed the code and methods in [15] except for MKD+ [16], for which we used the same configuration as in [16]. For IEMOCAP, we followed the optimizer, learning rates, and stopping criterion in [14]. For MKD, the parameters used in the experiments were $\lambda_{m}\in \left\{0.001,0.1,1,1.5,4.5,5,8\right\}$ and $T_{m}\in \left\{0.001,0.01,0.05,1,3.7\right\}$, depending on the database and combined approaches. We first crudely determined the parameter values using a grid search for a wide range of parameters, and then we adjusted them one-by-one to optimize the performance for the validation set for IEMOCAP and the test set for CREMA-D. The accuracy (ACC) and unweighted accuracy (UA) were used as evaluation metrics for CREMA-D and IEMOCAP, respectively, as in many previous works on the corresponding databases. ACC is the ratio of the number of correct predictions and test samples. UA is an average of the accuracies for individual emotional classes.

### 3.2. Results

Table 1 summarizes the performance of the MER systems and the number of parameters needed in the inference phase for CREMA-D and IEMOCAP. It is noted that this work focused on utterance-wise emotion recognition for six emotions for CREMA-D and seven emotions for IEMOCAP, while some other works focused on four-emotion classification or emotion recognition in conversation. In Table 1, the highest accuracy (ACC) and unweighted average (UA) values for each dataset are shown in bold. We can see that the adoption of the proposed MKD improved the performance of not only the original MER models but also the MER models with the previously proposed OGM-GE [15], PMR [16], MMCosine [17], and All-sum [26]. The best ACC for CREMA-D was 69.3%, which was achieved when the proposed MKD was combined with MMCosine loss, while the best UA of 68.7% was obtained for IEMOCAP when the ensemble of unimodal models and the MER model with MKD was employed. Although the performance improvement achieved with All-sum (MKD) over the best previously proposed method, All-sum [26], for IEMOCAP was not very high, it was statistically significant with a *p*-value of 0.037. All three previously proposed approaches that deal with optimization imbalance phenomenon improved the performance of MER on CREMA-D, and MMCosine [17] performed the best. However, MMCosine did not improve the performance on IEMOCAP, although we tried several fusion methods and achieved the best performance with concatenation fusion. This result may have been due to the modality imbalance for IEMOCAP being less severe, as can be seen in Table 2, and thus ignoring the magnitude of the weighted embedding for each modality would have resulted in information loss rather than modality balancing. In contrast, the ensemble approach denoted All-sum [26] produced less of a performance improvement than these three approaches on CREMA-D, and the performance of the ensemble of MKD with unimodal models was inferior to that of MKD. As can be seen from the performance of the unimodal models on CREMA-D in Table 2, the visual cues in CREMA-D were much weaker than the audio cues, which led to the poor performance of the simple ensemble of audio and visual modalities. MWCE and Self-KD [34] improved the MER performance on both of the databases but not as much as MKD. We confirmed that the performance improvement of the proposed MKD was not just due to the additional loss function for unimodal encoders or to the KD with soft labels.

Additionally, we checked if MKD actually mitigated the optimization imbalance phenomenon by examining the performance of each unimodal encoder in the MER model by attaching a linear classifier, as in [35]. Specifically, we froze the unimodal encoders in the MER models and trained a linear classifier for each of the unimodal encoders to identify emotional classes. Table 2 shows the ACCs (%) and UAs (%) of the unimodal models trained with a single modality and unimodal encoders in the MER model with and without MKD. It can be seen that each of the unimodal encoders in the MER model without MKD was not as good as the unimodal models for all cases, while the unimodal encoders in the MER model with MKD performed better than the unimodal models by utilizing both the KD from the unimodal models and the gradient affected by other modalities.

Table 3 presents the ablation study results for MKD in which the modality-wise knowledge distribution was applied to a subset of the unimodal encoders. The experimental results show that the performance was improved by applying MKD for each of the unimodal encoders one-by-one on both of the databases and for every modality. The performance improvement achieved by adding MKD for one more modality was almost the same for any modality in the experiment with IEMOCAP, although the performance of the unimodal pre-trained models was not the same.

## 4. Conclusions

In this paper, we propose a MER method using modality-wise knowledge distillation that utilizes pre-trained unimodal emotion recognition models to overcome the optimization imbalance phenomenon. In the proposed method, each unimodal encoder in the MER model is trained to produce similar logits with an extra linear classifier for the logits than a pre-trained unimodal model produces, while the whole MER model including unimodal encoders is also updated with the same minibatch using the loss for the MER. By guiding the unimodal encoders within the MER model using pre-trained unimodal models, each unimodal encoder can be trained well even though another unimodal encoder provides much more useful information for MER at a certain point in the training process. Experimental results showed that MKD outperformed previous approaches for addressing the optimization imbalance phenomenon, and the combination of MKD and those techniques could further improve the performance. The best performance was obtained when MKD was combined with MMCosine loss on the CREMA-D datase, and when using the ensemble of MER with MKD and unimodal models on the IEMOCAP dataset. Further investigation is needed to find out why different combinations are more effective on different databases. Future works include the application of MKD with more MER models and multimodal classifiers in other domains.

## Figures and Tables

**Table 1:** Performance of multimodal emotion recognition and the number of parameters for the proposed and comparison systems on CREMA-D and IEMOCAP databases.

Method	CREMA-D		IEMOCAP	
	#Param	ACC (%)	#Param	UA (%)
Multimodal	22.4M	55.9	1.6M	64.20
OGM-GE [15]	22.4M	62.2 *	-	-
PMR [16]	22.9M	61.8 *	-	-
MMCosine [17]	22.9M	66.4 *	1.6M	61.80
Uni-sum [26]	22.4M	55.9	1.6M	63.60
All-sum [26]	44.7M	60.3	3.3M	68.00
MWCE	22.4M	60.8	1.6M	65.70
Self-KD [34]	22.4M	60.3	1.6M	64.40
MKD	22.4M	67.7	1.6M	67.50
MKD+ [15]	22.4M	68.4	-	-
MKD+ [16]	22.9M	67.9	-	-
MKD+ [17]	22.4M	**69.3**	1.6M	66.90
All-sum (MKD)	44.7M	67.1	3.3M	**68.70**

* indicates the result from the original work.

**Table 2:** Modality-wise emotion recognition accuracies for unimodal models and unimodal encoders in multimodal models with and without MKD attached to linear classifiers.

Method	CREMA-D		IEMOCAP		
	Audio	Visual	Audio	Visual	Text
Unimodal	57.5	27.3	45.1	53.2	51.3
Multimodal	57.0	18.6	43.2	50.8	50.6
MKD	62.5	29.2	45.7	54.7	52.0

**Table 3:** Performance of multimodal emotion recognition with and without modality-wise knowledge distillation for individual unimodal encoders.

Audio	Visual	Text	CREMA-D	IEMOCAP
**✗**	**✗**	**✗**	55.9	64.2
**✓**	**✗**	**✗**	63.5	66.1
**✗**	**✓**	**✗**	62.9	66.1
**✗**	**✗**	**✓**	-	65.9
**✓**	**✓**	**✗**	67.7	66.4
**✓**	**✗**	**✓**	-	66.4
**✗**	**✓**	**✓**	-	66.2
**✓**	**✓**	**✓**	-	67.5

## Data Availability

Datasets mentioned in this paper can be downloaded using the following links: CREMA-D https://github.com/CheyneyComputerScience/CREMA-D (accessed on 25 August 2025), IEMOCAP https://sail.usc.edu/iemocap/ (accessed on 25 August 2025).
